# Dr. Alder's Proofs of the Proximate Causes of Diseases from the Practice of Physic

**Published:** 1810-02

**Authors:** Thomas Alder


### Dr. Alder's *Proofs of the proximate Causes of Diseases from the Practice of Physic.*

( In Continuation. )

Opium. It seems past doubt that opium has done good in the hot fit of an intermittent; and how are we to account for this on any system? We certainly cannot on the Brunonian: but I *by no means* receive that system; I believe it is *full* of error. It is most probable that opium makes the arterial system propel the blood more effectually to the surface of the body, while the relaxation which soon follows, allows it to send off a considerable quantity of its constituent parts by the excretories. Conformably to this, we find, that when opium has done good in the hot fit, its employment has soon been followed by an universal and most copious sweat, which it is clear must relieve the internal parts greatly of their congestions.

Wine. Wine has not that tendency to relax the excretories which opium has, and of consequence does not always seem proper where opium is. It however gives firmness of tone to the arterial system, and by so doing, when the excretories are not shut up, it is in many cases of fever, &c. of considerable service. But we do not in general find wine is proper in bad fevers for removing debility, or in many other fevers, till after the disease has got a turn; that is, till the bowels have been opened, and the blood flows tolerably freely from the veins into the arteries, while the excretories are not at the same time constricted by any inflammatory diathesis. Opium is usually made to precede it, and this takes off any inflammatory diathesis in the arteries and constriction in the excretories. I will not say that wine is useless in all other cases and states of fever than these, because it is given with success where there is any gangrene in the system, or where there is much simple debility, and in some other instances, when it is not always clear whether these states are present or not, and where indeed we must often use it if they are, though afterwards in due time we seek to remove them. And, indeed, in some cases of debility, it will itself make the respiration freer, and must be given for this purpose as well as (which in part follows from this) to make the arterial system stronger; but I mean that the above states should, I think, very often be inquired into, when there is any doubt about giving wine.

Every body knows that wine is good to remove *simple* debility, debility, whether this be the effect of a preceding disease or not; but it is *diseased debility*, that is, a debility which forms a constituent part of some disease which occasions difficulties. Debility uniformly follows an impeded pulmonary transmission, and is greater as this is so. This impeded pulmonary transmission, may be with a general check of the whole vascular system (as often happens in tetanus); or it may be with great congestion in the lungs (which almost always is the case in fever); the congested vessels too may be torpid, they may be irritated, or they may be inflamed. In almost all fevers they are torpid for some time at first, but in intermitting fevers most so. In some remittents and ardent fevers very little, and sometimes scarcely at all. In mild continued fevers they continue in a state of irritation a long time; but in many bad fevers particularly in bad remittents, they soon become more or less inflamed. Now debility in all these states must be removed by agents, suitable to the state of the lungs; and wine frequently is not that agent; bleeding often is: but these remedies are not always opposites. Again, when any other vital organ is congested from this disordered state of the lungs, debility may be great, and yet wine will be improper; bleeding often must be used. When the brain is much congested from this cause, an apoplectic state or tendency usually comes on; and this produces a great debility, which we know is not to be relieved by wine.

In cases where depressing passions chiefly act to check the respiration, impede the circulation through the lungs, and cause a febrile state and debility, wine and opium, with pure air and amusements, may give much relief, and these passions often have great share in producing sporadic fevers.

Pure Air. This is the best preventive for fevers we have, and in many cases is the best remedy. But it is evident it cannot with any propriety be used when the congestion on the lungs has gone on to inflammation, or indeed to much irritation, till after this inflammation or irritation shall have subsided.

In the torpid state of the lungs, which occurs in a cold fit, it appears very proper, and has from a few trials been found so. In the sweating stage, and in intermissions and perfect remissions, it seems very proper. And in the latter end of continued fevers, or at least when all great irritation has subsided in the lungs, it appears equally proper, and has been proved so in one or two trials. But, in mentioning trials I have been deficient, for I alluded only to artificial

*artificial pure air;* and the *natural* has been used in all these cases, in many thousand instances, with great success, while the artificial, in some of them, could be of more use, as it could be made stronger; though the natural in other cases is best as it is always applied, and from being weaker, is not so likely to cause unhealthy irritation.

The mode of action of pure air is peculiarly explicable on my principles, both in doing good and harm; its action either way cannot be explained otherwise than by them.

The Bath. In some cases the warm bath is of service, in others the tepid, and in others the cold. In those dreadful internal congestions (before demonstrated) which so quickly run into inflammation, bleeding and the warm bath may be of use; but the cold cannot, nor wine either, let heat and debility be as great as they may.

The cold bath, like wine, is most suited to mild fevers, and I believe in such, when used according to Dr. Currie's directions, it is a most excellent remedy.

It prevents the blood from hurrying up to the lungs by the veins; diminishes the expansion of this fluid, which is caused by heat; takes off that constriction from the excretories (which was caused by heat (from which perspiration is renewed); gives tone to the vascular system (which, now the excretories are open, is the more useful), and it takes off morbid irritability, and that inquietude of mind which arises from the morbid state which existed previous to its application. Finally, by all these ways it greatly relieves the lungs.

---

§. Some other remedies might be noticed, but I believe their *modus operandi* will not now in general be very difficult on the preceding principles. *Smoaking tobacco,* as a preventive of epidemics, is the only one which now occurs to me, that appears to be so. It certainly has been respectably recommended; Diemerbroeck supposes he preserved himself from the plague by its means.

When I was at Cambridge, a short time in the winter 1798-9, several young men there, met in the evening in a convivial party and smoked their pipes, as they said, to prevent catching a fever, which at that time had occasioned several to leave St. John's, and go home; and as they all escaped fever, they might impute their exemption to the use of tobacco, just as Diemerbroeck did: but the fact was, those who escaped bore the proportion of three hundred to one to those who were seized.

The smoke of tobacco, however, is a most powerful agent; and I can readily admit it is efficacious in preventing some diseases, epidemic, endemic, or sporadic: I mean those of an inflammatory nature, whether they arise altogether from catching cold, or from cold joined with bad air. And many epidemics (even many examples of "the plague," have been much of this kind. Its general use in Holland is supposed (and I think with justice) to have arisen from the people in that country finding that it considerably defended them from injuries from the noxious moisture of the climate; though Tulpius says, notwithstanding, that (which is somewhat in opposition to more recent declarations) most of the bodies which he has opened there, have had ulcers in the lungs. But I do not think it appears, that smoking tobacco is of any use against such fevers, as have nothing of a catarrhal or phlegmonic diathesis joined with them; or (of consequence) against a passive pulmonary congestion; or bad air uncombined with cold moisture, or simply cold. But, on the contrary, I see no reason to suppose it may not often be in some such cases in some degree prejudicial; though on many occasions it will drive the fluids to the surface of the body, and make pure air afterwards have a more pleasing effect.

I have tried the smoke of tobacco upon myself in two opposite states. I tried it once when I had an impeded pulmonary transmission without any inflammatory diathesis, and it made it much worse; it made my respiration and pulse almost cease; and the anxiety and oppression at the præcordia, and the debility, greater. But I have tried it several times to prevent inflammatory affections of the mouth, face, throat, and lungs, with very signal success; and I can recommend it in such cases as a most efficacious article, and the best which I know of. It is not too late to use it, when these affections (except pneumonia) are coming on, and in myself I have removed them by it. Last spring I was seized with an inflammatory affection on one side of my mouth, face and neck, extending to the ear, greatly exacerbated every evening, and then attended with the most excruciating pain. Pains in the face had been slightly epidemic all winter, as well also as a mild influenza. I used laudanum and emetic tartar, with a little temporary relief for six weeks, all that time keeping in the house, and keeping my head wrapt up. At last I had recourse to smoking tobacco on the pained side, till it made me sick, and for the first two or three times acted as an emetic. I particularly used it in an evening, and took tea before it, and a bason of hot water gruel not long after it, and then went to bed. A copious sweat soon broke out, out, and continued very long; I slept better, and in a few days was quite cured of the pains. When they recurred (which they often did) I always found smoking, till I was a little faint, completely removed them, and particularly if I took some warm simple liquid, both before and after. I have been rather minute in the description of this case, as it shews, I conceive, the efficacy of the smoke of tobacco, both in preventing one kind of epidemic (as well as endemic and sporadic diseases) and also in removing some of those excruciating pains in the face, for which the learned Dr. Heberden confessed he could find no remedy.

The action of tobacco in these cases is by relaxing the inflammatory state of vessels, and drivin gcongested fluids to the surface.

§. If I have thus explained the operation of medicines in a just way, according to my theory, I conceive the *practice of physic* affords confirmation of its truth. It has not been here within my province to suggest new remedies or preventives; I had only to ascertain (which by the bye was no easy matter) what were good practices, and what were bad, and then to shew *why* they were so. I am induced, however, to notice, that I think my theory suggests one new remedy, which, *though* simple, seems to me to be of general applicability, and very probably of considerable efficacy, though it may, till reflected on, appear very trivial; I mean, laying much on the left side, in order to facilitate the entrance of blood into the arteries, and particularly when venæsection is used; as this most probably would prevent syncope, which in this case would be highly disadvantageous or dangerous. I say most probably, but I am of opinion, that if bleeding was *proper*, it most certainly would; while at the same time it would greatly impede, and perhaps prevent the pulmonic congestion from forming again, which otherwise might happen in a few minutes; and probably, particularly if the patient was erect, or on the right side, to a greater extent than before; the faintness which many people are apt to suffer from losing only a small quantity of blood, tending to make the pulmonic vessels more inirritable and relaxed.

When speaking of any particular case, it often is exceedingly difficult to ascertain the best practice for it; but how much more difficult must it be to treat of remedies with any certainty and precision applicable to all cases? And supposing a person do this, he will yet be very liable not to be well understood, or misunderstood. I have spoken spoken of fevers *generally*; and I persuade myself that I have found it proved from extensive practice, that neither wine nor the cold bath is a general remedy for fever, and that bleeding is; while, at the same time, the cold bath and wine are most excellent remedies for the common fevers of Britain, and bleeding is commonly unnecessary, and often would be prejudicial; but if we take an extensive view, we shall find the common fevers of Britain make an exceedingly small proportion of fever-cases in general.

*Proofs from the general Causes of Diseases and of Fevers.*

The causes of fevers are the causes of diseases in general. The most general and the most powerful are bad air, and pain, mental or corporeal; though a disordered intestinal canal, however induced, has great effect. Under the term "bad air," I include any kind of atmosphere not sufficiently pure, as one too pure, I think, is no where to be found; whether the impurity of it arise from moisture, from the results of putrefaction, fermentation, respiration, or combustion, or from terrestrial expirations, &c. and whether it be applied sporadically, endemically, or epidemically, singly, or in several of these ways at once, and of one kind only, or of several kinds together. But I have some grounds to hope, that neither epidemic, endemic, nor sporadic kind of bad air, in general, produces much disease by itself; that is, that one of them, in general, either is not sufficiently concentrated, or is not applied a sufficient length of time to produce much disease, without being effected by one or both the other kinds, or by some other cause. Thus we generally find, that very unwholesome bed-rooms, or sitting rooms, houses, streets, towns, districts, and countries, often do not suffer much till an epidemic state of the air comes; and on the other hand we find, that during an epidemic state of the air, those who live in cheerfulness and in dry airy situations and apartments, commonly escape. There have been two or three instances in which this did not *appear* to be the case: but in one of these cases my observation, clearly, certainly is applicable, whatever it may to some *appear* to be, as the bad air in this case was light, and attacked elevated situations, in preference to low ones; and, in the other cases it most probably is so, as a light kind of bad air is a term which conveys nothing absurd. A great part of bad air, however, commonly is moisture, either directly from evaporation, from the ground, or from dews or clouds falling upon the the ground; and consequently bad air usually is low: it often is so low as to affect most the beasts which graze, and wild beasts in dens, and little animals and reptiles: though on other occasions, it has reached so high as to affect the birds.

THOMAS ALDER.

( To be continued. )

